# Association of Immune Cells, Inflammatory Cytokines, and Lung Cancer: A Mediating Mendelian Randomization Study

**DOI:** 10.1155/mi/3834641

**Published:** 2025-11-17

**Authors:** Lang Li, Ying Zhu, Chenchong Zhao, Yinuo Tangwu, Lvyuan He, Gaochen Lan

**Affiliations:** ^1^Department of Hematology, Jinhua Hospital of Traditional Chinese Medicine Affiliated to Zhejiang Chinese Medical University, 438 West Shuangxi Road, Jinhua 321017, China; ^2^Department of Pneumology, Jinhua Hospital of Traditional Chinese Medicine Affiliated to Zhejiang Chinese Medical University, 438 West Shuangxi Road, Jinhua 321017, China; ^3^Department of Oncology, The Second Affiliated Hospital of Fujian Medical University, 950 Donghai Street, Quanzhou 362018, Fujian, China

**Keywords:** causal relationship, immune cell, inflammatory cytokine, lung cancer, mendelian randomization

## Abstract

**Background:**

Lung cancer oncogenesis involves interactions between immune cells and inflammatory cytokines. However, their causal relationships and mediating mechanism remain unclear.

**Methods:**

Using a mediating Mendelian randomization (MR) approach, we analyzed genome-wide association study (GWAS) data for 731 immune-cellular phenotypes, 41 cytokines, and eight related lung cancer phenotypes.

**Results:**

*BAFF-R* expression in IgD^+^ CD24^+^ B cells was positively associated with lung adenocarcinoma (LUAD) risk (odds ratio [OR], 1.0168 [95% confidence interval [CI], 1.0006–1.0332]), mediated by macrophage migration inhibition factor (MIF) with a mediation proportion of 14.2% (95% CI, 0.00007–0.0046). *CD39* expression on secreting CD4 Tregs was related to an increased risk of small-cell lung cancer (SCLC) (OR, 1.0306 [95% CI, 1.0006–1.0616]), mediated by interleukin (IL)-2 and IL-6 with mediation proportions of 8.43% (95% CI, 0.00005−0.005) and 5.1% (95% CI, 0.00006–0.003), respectively. *CD28* expression on CD39^+^ CD4^+^ T cells was positively correlated to lung squamous cell carcinoma (LUSC) risk (OR, 1.0335 [95% CI, 1.009–1.0586]), inversely mediated by IL-16 (95% CI, −0.01 to −0.0003).

**Conclusions:**

These findings reveal the associations between immune cells, inflammatory cytokines, and lung cancer risk, providing insights into cancer diagnosis and treatment.

## 1. Introduction

Lung cancer remains the leading cause of cancer-related mortality, accounting for 18.0% of the global cancer-related deaths in 2020 [1]. It is broadly classified as small-cell lung cancer (SCLC) and non-SCLC (NSCLC), with the latter encompassing lung squamous cell carcinoma (LUSC) and lung adenocarcinoma (LUAD) [2]. Smoking and a family history of disease (including illness in the father, mother, or siblings) are known risk factors. Although the treatment modalities, such as surgical resection, radiotherapy, chemotherapy, and targeted therapies have greatly extended the survival time, the 5-years post-diagnosis survival rate remains between 10% and 20% in most countries [3]. The poor prognosis is exacerbated by the rapid progression of disease during the asymptomatic phase, often leading to delayed diagnosis and treatment. Therefore, identifying of novel biomarkers to assist in cancer diagnosis and treatment is crucial.

Lung cancer oncogenesis involves interactions between immune cells and inflammatory cytokines that can act as biomarkers [4]. Immune cells play dual roles in cancer immunity. For instance, CD4^+^ T and CD8^+^ T cells prolong survival, whereas plasmacytoid dendritic cells (DCs) and mast cells promote tumor progression [5–9]. Regulatory T cells (Tregs) disrupt the balance of helper T cells (Th1/Th2), facilitating tumor progression [10, 11]. Conversely, conventional DC1s promote Th1 cell polarization and enhance antitumor response [12]. Inflammatory cytokines link the immune cells and perform dual functions in cancer immunity. For example, interleukin (IL)-10 is produced by various leukocytes, including DCs, macrophages, natural killer cells, and B cells [13–15] and can suppress antitumor immunity by inhibiting antigen presentation by DCs [16, 17]. However, IL-10 also enhances CD8 T cell infiltration and memory responses, promoting antitumor immunity [18, 19]. The complex associations among immune cells, cytokines, and cancer compromise the reliability of biomarkers in the clinical practice. Therefore, understanding the causal relationships and mediating mechanisms of immune cells and inflammatory cytokines in lung cancer may provide a basis for their application in cancer diagnosis and treatment.

Mendelian randomization (MR) is a robust epidemiological tool for causal inference that utilizes genetic variants as instrumental variables (IVs) to explore the causal relationships between the exposures and outcomes. MR minimizes the biases from confounding factors and reverse causality [20]. In the present study, we hypothesized that specific immune cells and cytokines causally mediate the development of various lung cancer subtypes. Utilizing a mediating MR analysis revealed the causal relationships and mediating mechanism under the immune cell, inflammatory cytokines, and lung cancer with its eight related phenotypes using the genome-wide association study (GWAS) data from European: three related to family history of disease, two related to smoking history, and three related to pathological classification, which may provide novel insights into lung cancer diagnosis and treatment.

## 2. Materials and Methods

### 2.1. Study Design

**Figure 1:**
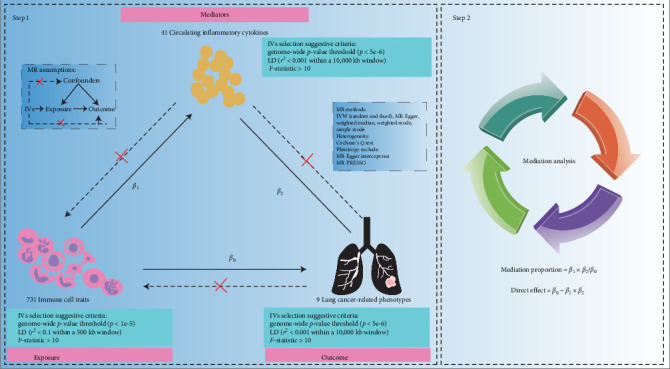
The study design of this study.

A mediating MR approach was employed to assess the mediation in the MR framework [21]. As shown in Figure 1, we first evaluated the associations between exposures and outcomes, exposures and mediators, and mediators and outcomes, respectively, using bidirectional two-sample MR analysis. We then screened the candidate immune-cytokine-cancer pathways. Second, mediation analysis was performed to evaluate the effect of cytokines on the candidate immune-cytokine–cancer pathways. Six MR methods were applied: inverse variance weighted (IVW) (fixed-effects), IVW (multiplicative random effects), and MR-Egger regression, simple mode, weighted mode, and weighted median using the TwoSampleMR R package (Supporting Information 1: Figure S1). A *p*  < 0.05 for the IVW method was regarded to the primary threshold of significance [22]. Results are reported as odds ratios (OR) per standard deviation (SD) change in genetically proxy phenotypes. This study adhered to the STROBR-MR guidelines [23] (Supporting Information 2: Table S1). The genetic mediation models adhered to the following assumptions: (1) no mediator–outcome confounding and (2) no exposure–mediator interaction.

### 2.2. Data Sources

GWAS summary data for immune cell traits and lung cancer-related phenotypes were obtained from the Integrative Epidemiology Unit GWAS project (https://gwas.mrcieu.ac.uk/) using the TwoSampleMR R package [24]. GWAS summary data for the 41 circulating inflammatory cytokines were downloaded from the University of Bristol Health Sciences database (https://data.bris.ac.uk/data/dataset/3g3i5smgghp0s2uvm1doflkx9x).

We included 731 immune cell traits (accession ID: GCST90001391–90002121) [25], including 118 absolute counts (AC), 389 median fluorescence intensities (MFI) reflecting cellular surface antigen abundances, 32 morphological parameters (MP), and 192 relative cell counts (RC). These traits include B cells, DCs, mature T cells, monocytes, myeloid cells, T cells, B cells, TBNK cells panel, and Tregs. The original GWAS was conducted on peripheral blood samples from 3757 Europeans, with genotypes for approximately 22 million single-nucleotide polymorphisms (SNPs) imputed using a Sardinian sequence-based reference panel [26]. Immune cellular phenotypes were measured using flow cytometry, and associations were adjusted for covariates (i.e., sex, age, and age^2^).

We included lung cancer with eight related phenotypes: three related to family history of disease (illness of father, mother, or siblings), two related to smoking history (lung cancer never smoked and ever smoked), and three related to pathological classification (SCLC, LUAD, and LUSC). These cohorts performed GWAS in the Europe with sample sizes ranging from 9857 to 423,523 and SNP numbers ranging from 7,438,318 to 10,894,596. (Supporting Information 1: Table S2).

Circulating inflammatory cytokines GWAS data were obtained from a study that investigated the associations between cytokines and growth factors [27]. The study included 8293 Finnish individuals from three cohorts: the Cardiovascular Risk in Young Finns Study (YFS) and the FINRISK surveys in 1997 and 2002.

### 2.3. IV Selection

For immune cell traits GWAS, SNPs with a *p* < 1 × 10^−5^ and a linkage disequilibrium (LD) threshold of *r*^2^ < 0.1 within a 500 kb window were selected [28, 29]. For lung cancer and circulating inflammatory cytokines, SNPs with a *p* < 5 × 10^−6^ and an LD *r*^2^ < 0.001 within a 10,000 kb window were selected [30, 31]. Where “kb” was the extent of regional LD, and the LD *r*^2^ was calculated based on 1000 Genomes Projects reference panel [32]. Clumping was performed using the PLINK software (version 2.00 α, https://www.cog-genomics.org/plink/2.0/). The F-statistic was used to evaluate the strength of each IVs; IVs with an *F*-statistic <10 were considered to weak [33] and excluded from the MR analysis. The *F*-statistic was calculated using the following formula:

  $$\begin{array}{c} F=\;R^{2}\times \frac{n-2}{1-R^{2}}, \end{array}$$

where *R*^2^ represents the proportion of variability explained by each IV and *n* is the sample size [34, 35]. *R*^2^ was calculated using the following formula:

  $$\begin{array}{c} R^{2}=\frac{\beta^{2}}{\beta^{2}+se^{2}\times n}\;, \end{array}$$

where *β* is the allele effect size, se is the standard error, and *n* is the sample size [36]. SNPs meeting the following criteria were retained as IVs: (1) strongly associated with exposures, (2) unrelated to confounders, and (3) affecting the outcomes via their effect on exposures [23].

### 2.4. Sensitive Analysis

Heterogeneity among IVs was assessed using Cochran's Q statistic, with a *p*  < 0.05 indicating heterogeneity. In such cases, the IVW multiplicative random effects method was used instead of fixed-effects as the primary method to avoid a false positive [37]. Horizontal pleiotropy was evaluated using the MR-Egger intercept test, with a *p*  < 0.05 indicating pleiotropy [38, 39]. The MR pleiotropy residual sum and outlier (MR-PRESSO) method was used to detect and correct for pleiotropy via the MRPRESSO R package, with a *p*  < 0.05 in the global test indicating its presence [40].

### 2.5. Mediation Analysis

The direct effect with immune cell traits on each lung cancer-related phenotypes was calculated via the following formula:

  $$\begin{array}{c} \text{Direct effect}=\beta_{0}\;-\beta_{1}\times \beta_{2}. \end{array}$$

The mediation proportions were calculated according to following formula:

  $$\begin{array}{c} \text{Mediation proportions}=\left(\beta_{1}\times \beta_{2}\right)\;/\beta_{0}, \end{array}$$

where *β*_0_ is the effect of immune cell traits on lung cancer-related phenotypes, *β*_1_ is the effect of immune cell traits on circulating inflammatory cytokines, and *β*_2_ is the effect of circulating inflammatory cytokines on lung cancer-related phenotypes. Standard errors and 95% CIs for mediation effects were calculated using the delta methods [41] in the RMediation R package [42]. *p*  < 0.05 was considered to significant. Cytokines meeting the following criteria were considered to mediators: (1) statistically significant in the mediation analysis and (2) previously reported to conform to molecular biological principles and exhibit clinical relevance [43].

### 2.6. Statistical Analysis

All data analyses were performed using the R software (version 4.3.1, https://cloud.r-project.org). Six MR methods were used to evaluate associations between exposures and outcomes. The *p*-values were adjusted via Benjamini– Hochberg method to control type I error.

## 3. Results

### 3.1. Selection of IVs

We identified 21,058 SNPs associated with 731 immune cell traits (*p* < 1 × 10^−5^, LD *r*^2^ <0.1 within a 500 kb window, and *F*-statistic >10). For 41 circulating inflammatory cytokines, a total of 229 SNPs met the criteria (*p* < 5 × 10^−6^, the LD *r*^2^ < 0.001 within a 10,000 kb window, and *F*-statistic >10). For lung cancer and its eight related phenotypes, a total of 231 SNPs were selected (*p* < 5 × 10^−6^, the LD *r*^2^ < 0.001 within a 10,000 kb window, and *F*-statistic >10) (Supporting Information 3: File 1–3).

### 3.2. Causal Effects of Immune Cell Traits on Lung Cancer-Related Phenotypes

**Figure 2:**
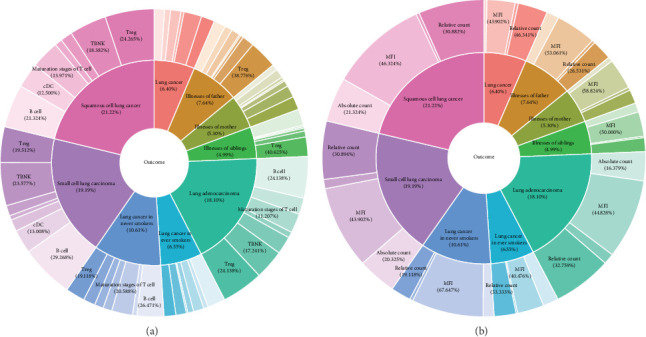
Causal effects of immune cell traits on lung cancer-related phenotypes. (A) Distribution of causal immune cell traits across cellular types (e.g., T cells, B cells, and myeloid cells) associated with nine lung cancer-related phenotypes. (B) Distribution of immune cell traits by functional categories (e.g., absolute counts, surface markers, and differentiation stages) across phenotypes.

A total of 395 immune cell traits exhibited causal effects on lung cancer and its eight related phenotypes, including lung cancer (6.4%), illness of the father (7.64%), illness of the mother (5.3%), illness of siblings (4.99%), LUAD (18.1%), SCLC (19.19%), LUSC (21.22%), lung cancer in never smokers (6.55%), and lung cancer in ever smokers (10.61%). These traits were distributed across different cell panels, including Tregs, cDCs, B cells, TBNK cells, and the maturation stages of T cells for the nine lung cancer-related phenotypes. (Figure 2A, B; Supporting Information 3: File 4–5).

### 3.3. Causal Effects of Immune Cell Traits on Circulating Inflammatory Cytokines

**Figure 3:**
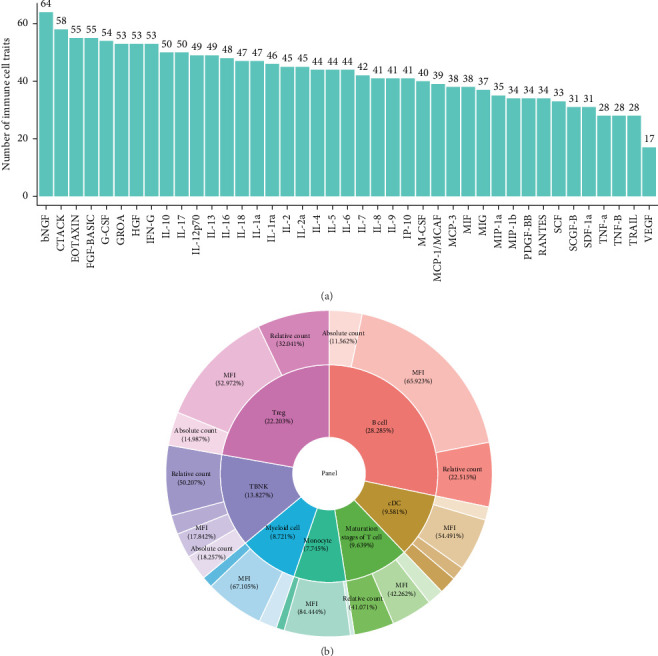
Causal effects of immune cell traits on circulating inflammatory cytokines. (A) Number of immune cell traits exerting causal effect on each of 41 circulating inflammatory cytokines. (B) Cellular type and functional category distribution of cytokine-associated immune cell traits.

A total of 594 immune cell traits showed a causal effect on 41 circulating inflammatory cytokines, with 17–64 traits being associated with each cytokine. These traits spanned seven cell panels: Tregs (22.203%), B cells (28.285%), TBNK (13.827%), maturation stages of T cells (9.639%), cDCs (9.581%), myeloid cells (8.721%), and monocytes (7.45%) (Figure 3A, B; Supporting Information 3: File 6–7).

### 3.4. Causal Effects of Circulating Inflammatory Cytokines on Lung Cancer-Related Phenotypes

**Figure 4:**
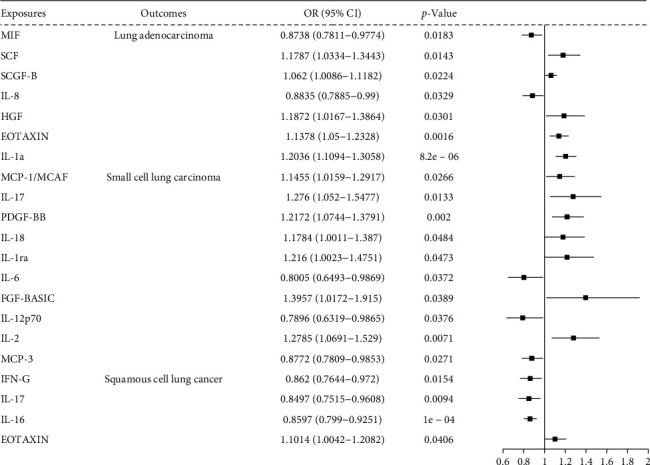
Forest plot depicts inverse variance weighted (IVW) mendelian randomization (MR) estimates for cytokine effects on lung adenocarcinoma (LUAD), small-cell lung cancer (SCLC), and lung squamous-cell carcinoma (LUSC).

Twenty circulating inflammatory cytokines exhibited causal effects in LUAD, LUSC, and SCLC. Seven circulating cytokines, including macrophage migration inhibition factor (MIF), stem cell factor (SCF), stem cell growth factor- B (SCGF-B), IL-8, hepatocyte growth factor (HGF), eosinophilic activating chemotactic factor (EOTAXIN), and IL-1a, were associated with LUAD. Ten cytokines, including monocyte chemoattractant proteins-1 (MCP-1/MCAF), IL-17. platelet-derived factor PDGF-BB, IL-18, IL-1ra, IL-6, fibroblast growth factor-basic (FGF-BASIC), IL-12p70, IL-2, and MCPs-1 (MCP-3), were associated with SCLC. Four cytokines, including interferon-G (IFN-G), IL-17, IL-16, and EOTAXIN, were associated with LUSC (Figure 4; Supporting Information 3 File 8–9).

### 3.5. Overall Associations of Candidate Immune-Cytokine-Cancer Pathways

**Figure 5:**
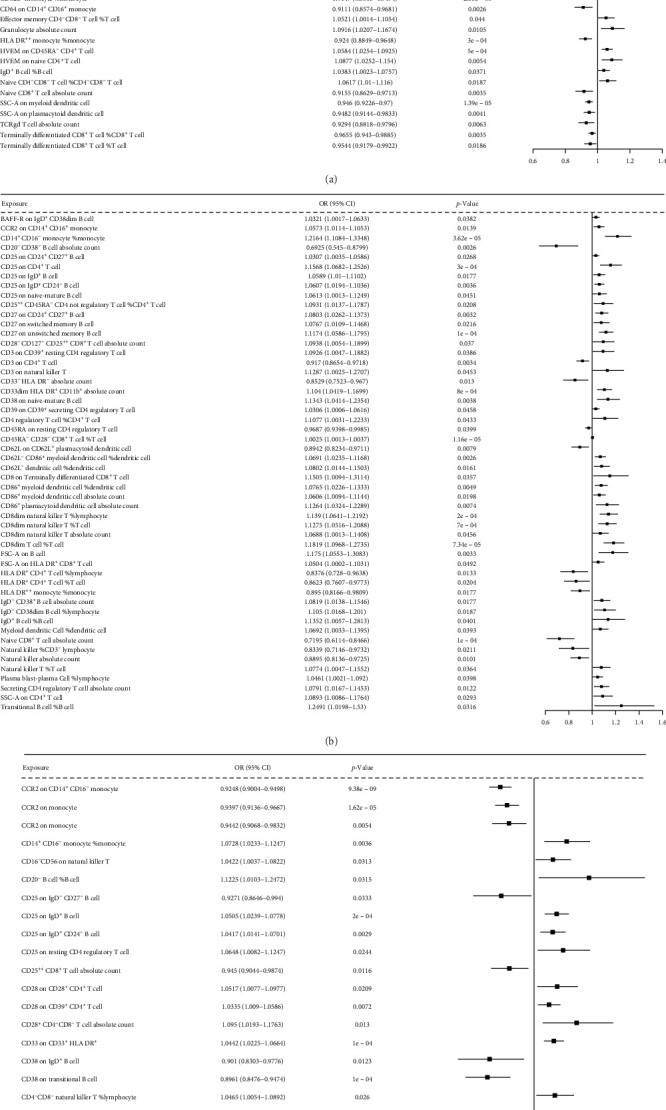
Forest plots depict IVW MR-derived causal estimates overall associations of candidate immune-cytokine-cancer pathways for LUAD (A), SCLC (B), and LUSC (C).

The risk of LUAD, SCLC, and LUSC risks are influenced by the immune cell traits through circulating inflammatory cytokine mediators.  Figure 5A shows the IVW MR results for LUAD, with 47 immune cell traits exhibiting causal effects mediated by six cytokines. Of these, 26 immune cell traits were positively related to LUAD risk (range of ORs, 1.0168 [95% CI, 1.0006–1.0332] for *BAFF-R* expression on IgD^+^ CD24^+^ B cell to 1.0004 [95% CI, 1–1.0007] for CD28^−^ CD8^+^ T cell %T cell), 21 immune cell traits negatively related to LUAD risk (range of ORs, 0.911 [95% CI, 0.857–0.968] for *CD64* expression on CD14^+^ CD16^+^ monocyte to 0.985 [95% CI, 0.971–0.999] for activated and resting CD4 Treg %CD4 Treg).  Figure 5B shows the 52 immune cell traits associated with SCLC via cytokines. Of these, 41 immune cell traits were positively related to SCLC risk (range of ORs, 1.0025 [95% CI, 1.0013–1.0027] for CD45RA^−^ CD28^−^ CD8^+^ T cell %T cell to 1.249 [95% CI, 1.0198–1.53] for transitional B cell %B cell), 11 immune cell traits were negatively related to SCLC risk (range of ORs, 0.6925 [95% CI, 0.545–0.8799] for CD20^−^ CD38^−^ B cell AC to 0.9687 [95% CI, 0.9398–0.9985] for CD45RA on resting CD4 Treg).  Figure 5C shows the 30 immune cell traits associated with SCLC through the 10 cytokines. Among these, 22 immune cell traits were positively related to LUSC risk (range of ORs, 1.001 [95%CI, 1–1.002] for CD45RA^−^ CD28^−^ CD8^+^ T cell %T cell to 1.1225 [95%CI, 1.0103–1.12472] for CD20^−^ B cell %B cell). Nine immune cell traits were negatively related to LUSC risk (range of OR, 0.8961 [95%CI, 0.8476–0.9474] for CD38 on transitional B cell to 0.9611 [95%CI, 0.9298–0.9936] for herpesvirus entry mediator on terminally differentiated CD8^+^ T cell) (Supporting Information 3: File 10).

### 3.6. Association Between Circulating Inflammatory Cytokine Mediators and Lung Cancer Outcomes in Candidate Pathway

**Figure 6:**
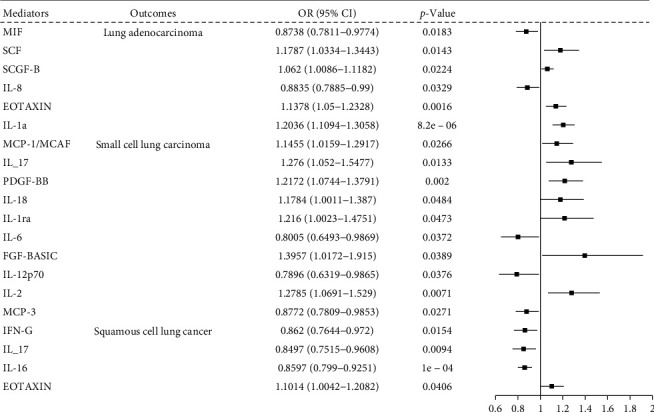
Cytokine-specific effects on lung cancer subtypes IVW MR estimates for casual associations between circulating inflammatory cytokines and LUAD, SCLC, and LUSC.

Figure 6 shows the associations between cytokine mediators and lung cancer outcomes. Six cytokines mediated the relationship between immune cell traits and LUAD. Of these, four cytokines including SCF, EOTAXIN, SCGF-B, and IL-1a, were positively associated with LUAD risk (range of OR, 1.062 [95% CI, 1.0086–1.1182] for SCGF-B to 1.2036 [95% CI, 1.1094–1.3058] for IL-1a), while two cytokines including MIF ([ORs], 0.8738 [95% CI, 0.7811–0.9774]) and IL-8 ([ORs], 0.8835 [95% CI, 0.7885–0.99]), were negatively related to LUAD risk. Ten cytokines showed mediating effects between immune cell traits and SCLC risk. Among these, seven cytokines including MCP-1/MCAF, IL-17, PDGF-BB, IL-18, FGF-BASIC, IL-1ra, and IL-2, exhibited positive mediating effect on immune cell traits to SCLC risk (range of OR, 1.18 [95% CI, 1.1784–1.387]) for IL-18 to 1.3957 [95% CI, 1.0172–1.915]) for FGF-BASIC, whereas IL-12p70, IL-6, and MCP-3 showed negative mediating effect on immune cell traits to SCLC risk (range of OR, 0.7896 [95% CI, 0.6319–0.9865] for IL-12p70 to 0.8772 [95% CI, 0.7809–0.9853] for MCP-3). The four cytokines demonstrated mediating effects on immune cell traits and LUSC risk. Of these, EOTAXIN exhibited positive mediating effect on immune cell traits to LUSC risk (OR, 1.1014 [95% CI, 1.0042–1.2082]), whereas INF-γ, IL-17, and IL-16 showed negative mediating effects on immune cell traits to LUSC risk (range of OR, 0.8497 [95% CI, 0.7515–0.9608] for IL-17 to 0.862 [95% CI, 0.7644–0.972] for INF-γ) (Supporting Information 3: File 11).

### 3.7. Associations of Immune Cell Trait Exposures and Circulating Inflammatory Cytokine Mediators in the Candidate Pathway

**Figure 7:**
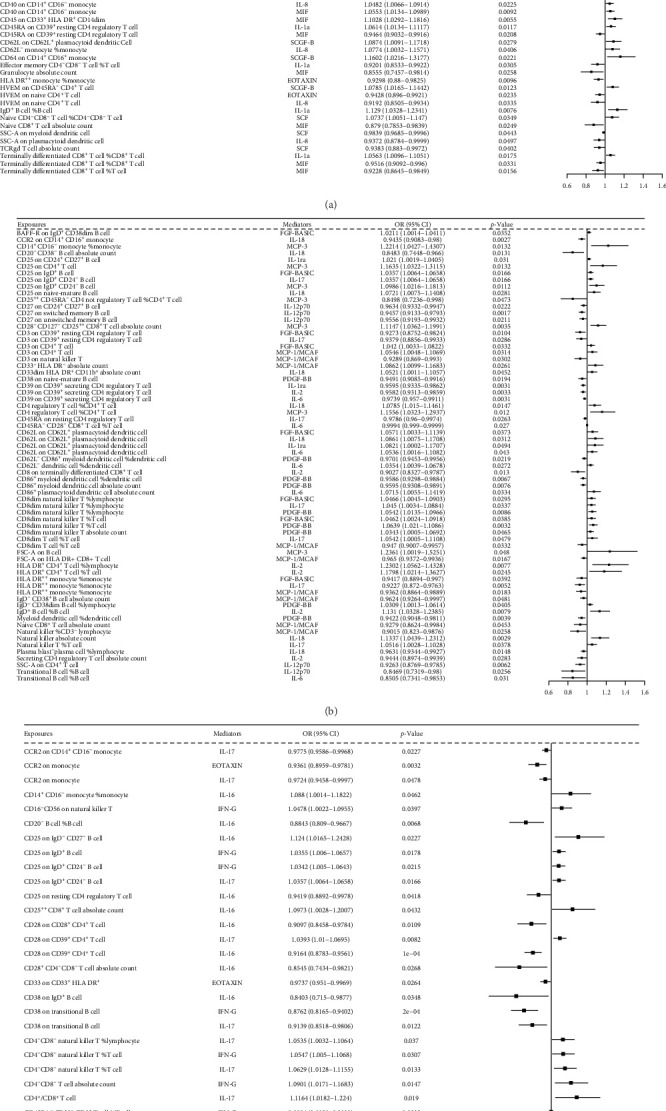
Immune-cytokine interactions in lung cancer subtypes IVW MR analysis of immune cell trait-cytokine mediator associations in LUAD (A), SCLC (B), and LUSC (C).

Figure 7A illustrates the association between immune cell traits and circulating inflammatory cytokine mediators in LUAD outcomes. A total of 47 immune cell traits causally affected LUAD risk through six cytokine mediators. Of these, twenty-eight traits exhibited negative associations with LUAD risk (range of ORs, 0.8555 [95% CI, 0.7457–0.9814] for granulocyte AC via MIF to 0.9546 [95% CI, 0.9134–1.1191] for SCC-A on myeloid DCs via SCF). Conversely, 19 traits demonstrated positive associations (range of OR, 1.0414 [95% CI, 1.0005–1.0839] for CD11c on myeloid DC via IL-1a to 1.2444 [95% CI, 1.0051–1.5407] for CD28^−^ CD8^+^ T cell %T cell via SCGF-B mediator). Figure 7B delineates these associations in SCLC. A total of 42 immune cell traits affected SCLC risk via 10 cytokine mediators. Of these, 20 traits were positively associated with SCLC risk (range of OR, 1.021 [95% CI, 1.0019–1.0405] for CD25 on CD24^+^ CD27^+^ B cell on IL-1a mediator to 1.2361 [95% CI, 1.0019–1.5251] for FSC-A on B cell on MCP-3 mediator). By contrast, 32 traits showed protective effects (range of ORs, 0.8469 [95% CI, 0.7319–0.98] for transitional B cell %B cell on IL-12p70 mediator to 0.9780 [95% CI, 0.96–0.9974] for CD45RA^−^ CD28^−^ CD8^+^ T cell %T cell on IL-6 mediator). Figure 7C shows the relationships in LUSC. Thirty immune cell traits mediated risk via the four cytokines. Seven traits were positively associated with LUSC (range of ORs, 1.0342 [95% CI, 1.005–1.0643] for CD25 on IgD^+^ CD24^−^ B cell on INF-γ mediator to 1.124 [95% CI, 1.0165–1.2428] for CD25 on IgD^−^ CD27^−^ B cell on IL-16 mediator). Twenty-four traits conferred protective effects (range of ORs, 0.8403 [95% CI, 0.715–0.9877] for CD38 on IgD^+^ B cell on IL-16 mediator to 0.999 [95% CI, 0.9989–0.9999] for CD45RA^−^ CD28^−^ CD8^+^ T cell %T cell on INF-γ mediator (Supporting Information 3: File 12).

### 3.8. Mediating Effects of Cytokine in the Association Between Immune Cell Traits and Lung Cancer

Mediation analysis based on MR estimated the proportion of causal effects mediated by inflammatory cytokines between immune cell traits and lung cancer phenotypes (Supporting Information 3: File 13). As shown in Table 1, MIF mediated 14.2% (95% CI, 0.00007−0.0046) of the causal pathway linking *BAFF-R* expression on IgD^+^ CD24^+^ B cells to LUAD risk. IL-2 mediated 8.43% (95% CI, 0.00005−0.005) of the association between *CD39* expression on CD39^+^ secreting CD4 Tregs and SCLC risk. IL-6 mediated 5.1% (95% CI, 0.00006−0.003) of the association. A negative mediation proportion was observed for the IL-16 (95% CI, −0.01 to −0.0003), indicating an inverse mediating role in the causal effect of *CD28* expression on CD39^+^ CD4^+^ T cells on LUSC risk (Table 1).

## 4. Discussion

The tumor immunity is regulated by an intricate cellular network, and cytokine-mediated intercellular communication plays a pivotal regulatory role. Using mediation MR analysis, we systematically investigated the mediating effect of 41 circulating inflammatory cytokines on the associations between 731 immune cell traits and lung cancer with its eight related phenotypes. These findings may serve as biomarkers and provided potential therapeutic targets for lung cancer. In the clinical practice, eliminating the immune cells, cytokines, and lung cancer cells may improve lung cancer.

**Figure 8:**
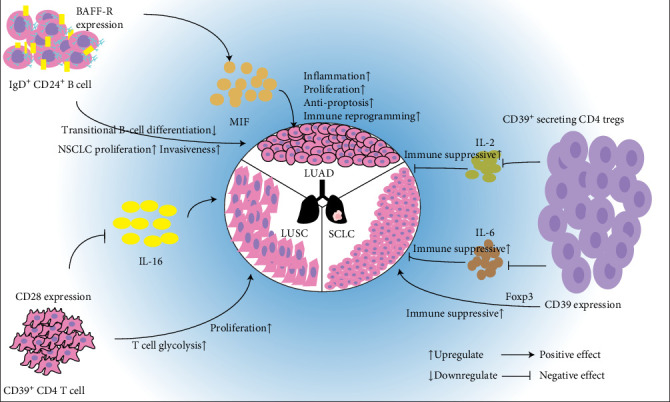
The model diagram of the immune cell, cytokine, and lung cancer relationships.

Our data indicated that the elevated *BAFF-R* expression on IgD^+^ CD24^+^ B cells increased the LUAD risk (OR, 1.0168 [95% CI, 1.0006−1.0332]), with 14.2% (95% CI, 0.00008−0.0046) of this effect mediated by MIF.  Figure 8 illustrates the mechanistic framework. *BAFF-R* critically regulates B-cell maturation; its overexpression impedes transitional B cell differentiation, leading to immunodeficiency characterized by B-lymphopenia and impaired humoral responses [44]. In vitro studies have demonstrated that BAFF-R signaling enhances NSCLC viability, proliferation, and invasiveness in A549 and H2030 cell lines [45]. MIF, a pleiotropic immune modulator, promotes protumorigenic processes, including inflammation, cell proliferation, antiapoptotic signaling, and immune reprograming [46]. Although co-expression and direct BAFF-R/MIF binding remains uncharacterized in LUAD, peripheral MIF levels amplify BAFF associated to increased LUAD risk, potentially through the synergistic modulation of tumor microenvironment dynamics, which aligns with molecular logic. Further investigation is required in this field. *CD39* expression on CD39^+^ secreting CD4 Tregs and exhibited a positive association with SCLC risk (OR, 1.0306 [95% CI, 1.0006–1.0616]), mediated by IL-2 and IL-6, with mediation proportions of 8.43% (95% CI, 0.00005–0.005) and 5.1% (95% CI, 0.00006–0.003), respectively. As a key ectonucleotidase in ATP/ADP–AMP–adenosine pathway [47], *CD39* maintains Tregs immunosuppressive function through Foxp3 (transcription factor forkhead box protein 3) stabilization [48, 49], which shortens the survival time of patients with SCLC [50]. IL-2 preforms an immunosuppressive effective by sustaining Tregs survival and activity, as evidenced by IL-2 genes deficiency in mice [51] and peripheral Treg depletion following IL-2 neutralization [52]. IL-6 enhances Treg proliferation in vitro [53], which also suppresses the immunity. *CD28* expression on CD39^+^ CD4^+^ T cell and raised LUSC risk (OR, 1.0335 [95% CI, 1.009–1.0586]), paradoxically counterbalanced by IL-16 through negative mediation (95% CI, −0.01 to −0.0003). CD 28 constimulation enhances T cell glycolysis to fuel proliferation [54]. IL-16 is a chemoattractant of CD4^+^ lymphocytes, which has rarely been reported in LUSC, and has demonstrated negative effects on multiple myeloma [55] by inducing cell proliferation [56] and protective effects against ovarian cancers [57] by promoting angiogenesis [58]. In the present study, IL-6 was demonstrated a positive effect on LUSC risk. This discrepancy may stem from tumor-type-specific IL-16 signaling. In the present study, many mediation proportions were <15%, and ORs were close to 1, suggesting that cytokines partially mediate effects on lung cancer, and distinct type of immune cells play limited roles in the lung cancer oncogenesis. Not all findings could be used as biomarkers and therapeutic target, and the *BAFF-R* expression on the IgD^+^ CD24^+^ B cell-MIF-LUAD pathway may be the most promising biomarker and will achieve clinical translation after in vivo and in vitro validation based on the literatures and statistical analyses.

Previous studies have still reported an association between immune cells and lung cancer. For example, Zhou et al. [29] performed a MR analysis to identify the causative role of immune cells in lung cancer and identified seven protective and 11 hazardous immune cell populations for lung cancer. However, they did not identify the possible mediators of these associations. Zhu et al. [59] explored the causal relationship between the immune cell-inflammatory factor axis and lung cancer using MR analysis. However, they did not describe the associations between distinct lung cancer subtypes. In the present study, we extend these studies and evaluated the mediating effects of circulating inflammatory cytokines on the associations between immune cells and lung cancer with its eight related phenotypes.

Our study has some limitations. Although MR analysis reduces the susceptibility to reverse causation and environmental confounding, residual bias from unmeasured pleiotropic pathways or horizontal pleiotropy (e.g., unmeasured behaviors and environmental factors, differential methods used to measure the phenotypes, and differential models used to perform GWAS analysis) cannot be fully excluded. The GWAS data were predominantly derived from European ancestral populations, which potentially limits the generalizability of ours results to non-European ethnic groups. Therefore, caution is warranted when extrapolating these findings to populations with distinct ancestral backgrounds. Owing to equipment limitations, we did not perform the ancestry harmonization or population structure adjustments, which also resulted in bias. The technical variability, interlab differences, and timing of sample collection for the flow cytometry used to measure the immune traits limit the application of these findings in the clinical practice. Although our MR framework supports causal inference, the precise biological mechanism underlying observed immune–cytokine–cancer interactions (e.g., BAFF-R/MIF crosstalk) requires experimental validation using preclinical models.

## 5. Conclusion

This study revealed the associations between immune cells, inflammatory cytokines, and lung cancer risk, providing new insights into cancer diagnosis and treatment.

## Figures and Tables

**Table 1:** The mediation proportion of mediators in the casual association between immune cell traits and lung cancer-related phenotypes.

Immune cell traits	Mediators	Outcomes	*β*_0_	*β*_1_	*β*_2_	Direct effect	Mediation effect (95% CI)	Mediation proportion (%)	*p*-Value
BAFF-R on IgD^+^ CD24^+^ B cell	MIF	LUAD	0.017	−0.047	−0.050	0.014	0.002 (7.62 × 10^−5^–0.0046)	14.20	0.03
CD39 on CD39^+^ secreting CD4 Tregs	IL-2	SCLC	0.3017	−0.043	−0.060	0.03	0.0025 (5.15 × 10^−5^–0.005)	8.43	0.039
CD39 on CD39^+^ secreting CD4 Tregs	IL-6	SCLC	0.3017	−0.026	−0.06	0.03	0.0015 (6.6 × 10^−5^–0.003)	5.1	0.035
CD28 on CD39^+^ CD4^+^ T cell	IL-16	LUSC	0.033	−0.088	0.06	0.038	-0.005 (−0.01 to −0.0003)	NA	0.034

*Note:* β_0_, total effect; *β*_1_, effect of exposures on mediators; *β*_2_, effect of mediators on outcomes.

Abbreviations: CI, Confidence interval; NA, not applicable.

## Data Availability

The datasets analyzed during the current study are available in the IEU GWAS project (https://gwas.mrcieu.ac.uk/) and University of BRISTOL health sciences database (https://data.bris.ac.uk/).

## Nomenclature

SCLC: Small-cell lung cancer
NSCLC: No small cell cancer
LUSC: Lung squamous cell carcinoma
LUAD: Lung adenocarcinoma
GWAS: Genome-wide association study
IEU GWAS: Integrative epidemiology unit genome-wide association study
API: Application programing interface
AC: Absolute counts
MFI: Median fluorescence intensities
MP: Morphological parameters
RC: Relative cell counts
SNPs: Single-nucleotide polymorphisms
LD: Linkage disequilibrium
IVs: Instrument variates
IVW: Inverse variance weighted
MR: Mendelian randomization
OR: Odds ratios
SD: Standard deviation
MR-PRESSO: Mendelian randomization pleiotropy residual sum and outlier
BMI: Body mass index
DCs: Dendritic cells
CI: Confidence interval
MIF: Macrophage migration inhibition factor
SCF: Stem cell factor
EOTAXIN: Eosinophilic activating chemotactic factor
HGF: Hepatocyte growth factor
SCGF-B: Stem cell growth factor-B
FGF-BASIC: Fibroblast growth factor-basic
IFN-G: Interferon-G
MCP-3: Monocyte chemoattractant proteins-3
PDGF-BB: Platelet-derived factor
CTACK: Cutaneous T cell-attracting chemokine
ATP/ADP–AMP–adenosine: Adenosine triphosphate/adenosine diphosphate–adenosine monophosphate
Foxp3: Transcription factor forkhead box protein 3
*BAFF-R*: B-cell activating factor-receptor.

## Supporting Information

Supporting Information 1 Additional file 1: Table S2. Information of lung cancer-related phenotype GWAS data sources used in this study. Figure S1. The schematic diagram for this work.

Supporting Information 2 Additional file 2: Table S1. STROBE-MR checklist.

Supporting Information 3 Additional file 3: File 1. List of IV with lung cancer. File 2. List of IV with 41 circulating inflammatory cytokines. File 3. List of IV with 731 immune cell traits. File 4. Causal effects of immune cell traits on lung cancer-related phenotypes. File 5. The results of sensitive analyses for the associations between 731 immune cell traits and lung cancer-related phenotypes. File 6. Causal effects of immune cell traits on circulating inflammatory cytokines. File 7. The results of sensitive analyses for the associations between 731 immune cell traits and 41 circulating inflammatory cytokines. File 8. Causal effects of inflammatory cytokines on lung cancer-related phenotypes. File 9. The results of sensitive analyses for the associations between 41 circulating inflammatory cytokines and lung cancer-related phenotypes. File 10. Overall association among genetically estimated candidate immune cell traits, circulating inflammatory cytokine mediators, and lung cancer outcomes. File 11. Association between genetically estimated candidate circulating inflammatory cytokine mediators and lung cancer outcomes. File 12. Associations between genetically estimated candidate immune cell traits and circulating inflammatory cytokine mediators. File 13. Mediation proportion and 95% CI of candidate immune cell-cytokine-cancer pathway.
