# Supplementary material for: Association of Immune Cells, Inflammatory Cytokines, and Lung Cancer: A Mediating Mendelian Randomization Study
Source: Mediators Inflamm. 2025 Nov 17;2025:3834641. doi: 10.1155/mi/3834641 (PMC12643680; doi:10.1155/mi/3834641)
Supplement: Supporting Information 1 — Additional file 1: Table S2. Information of lung cancer-related phenotype GWAS data sources used in this study. Figure S1. The schematic diagram for this work. [file 3834641.f1.docx]

**Table S2.** Information of lung cancer-related phenotype GWAS data source used in this study.

| GWAS-ID | Phenotypes | Sample size | Case | Control | Population | SNPs |
| --- | --- | --- | --- | --- | --- | --- |
| ukb-a-54  ukb-b-14521 | Lung cancer  Lung cancer father | 337159  401624 | 190  37446 | 336969  364181 | European  European | 10894596  98518667 |
| ukb-b-20176 | Lung cancer mother | 423525 | 17566 | 405692 | European | 9851867 |
| ukb-b-15826 | Lung cancer siblings | 361586 | 8199 | 353387 | European | 9851867 |
| ebi-a-GCST004747 | Lung cancer never smoker | 9859 | 2355 | 7505 | European | 7993812 |
| ebi-a-GCST004749 | Lung cancer ever smoker | 40187 | 23223 | 16964 | European | 7924801 |
| ieu-a-988 | Small cell lung carcinoma | 23371 | 2791 | 20580 | European | 7438318 |
| ieu-a-984 | Lung adenocarcinoma | 65864 | 11245 | 54619 | European | 10345176 |
| ieu-a-989 | Squamous cell lung cancer | 62467 | 7704 | 54763 | European | 10341529 |


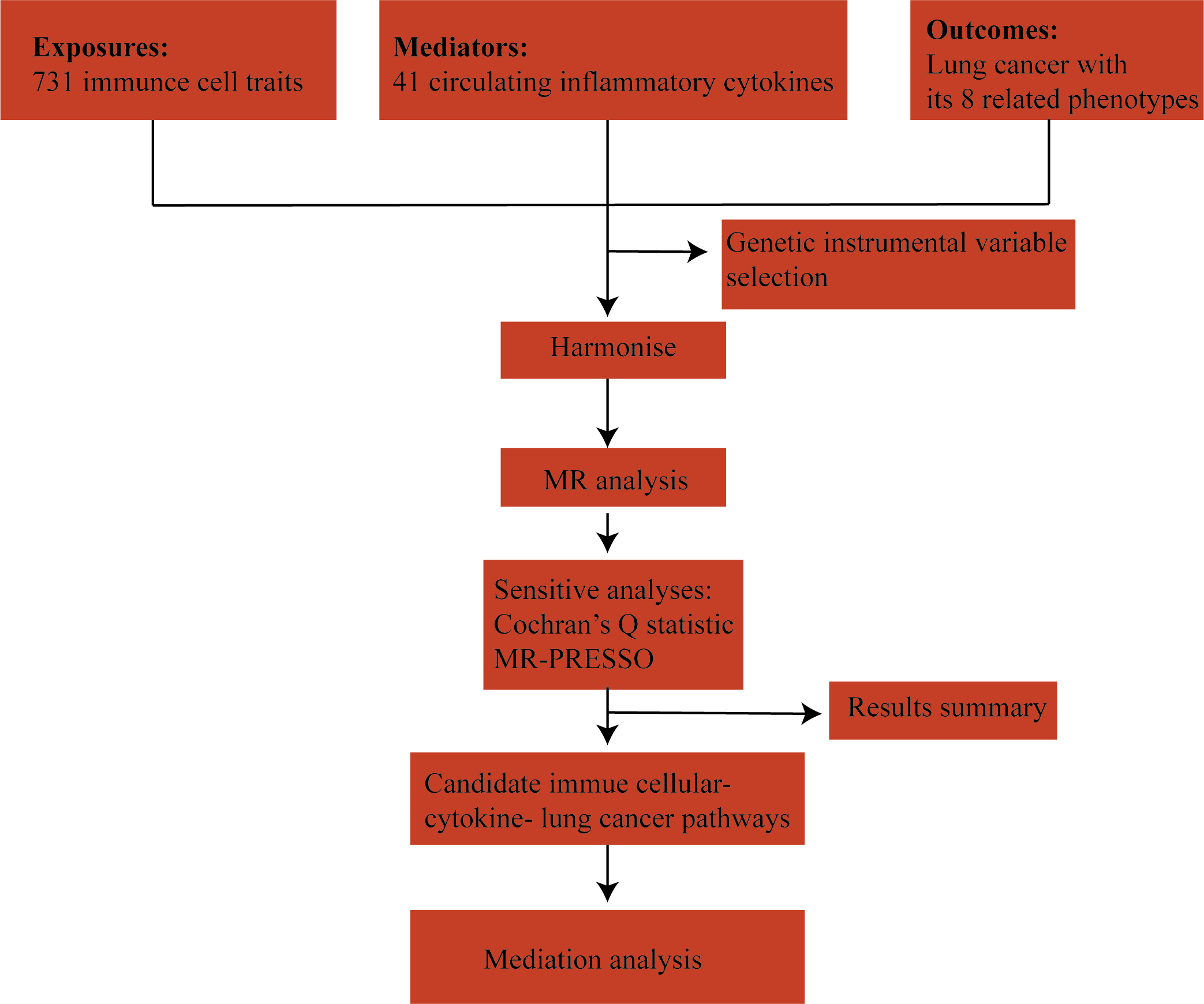


**Fig S1.** The schematic diagram for this work.
