# Supplementary material for: Association of Immune Cells, Inflammatory Cytokines, and Lung Cancer: A Mediating Mendelian Randomization Study
Source: Mediators Inflamm. 2025 Nov 17;2025:3834641. doi: 10.1155/mi/3834641 (PMC12643680; doi:10.1155/mi/3834641)
Supplement: Supporting Information 2 — Additional file 2: Table S1. STROBE-MR checklist. [file 3834641.f2.docx]

# Table S1 Strobe-MR checklist

| **Item** | **Complete/location** |
| --- | --- |
| 1. **Title and Abstract:** "Mendelian randomization" is named both in the title and the abstract | Complete |
| **Introduction** |  |
| 1. **Background:** Explain the scientific background and rationale for the reported study. Is causality between exposure and outcome plausible? Justify why MR is a helpful method to address the study question. | The scientific background and rationale for our research is described in the “Introduction” in the first two paragraphs. We then move on and describe the importance of MR in paragraph three. |
| 1. **Objectives:** State specific objectives clearly, including pre-specified causal hypotheses (if any). | Objectives are clearly described at the end of the 3^rd^ paragraph in the Introduction. |
| **Methods** |  |
| 1. **Study design and data sources:** Present key elements of study design early in the paper. Consider including a table listing sources of data for all phases of the study. For each data source contributing to the analysis, describe the following:   a) Describe the study design and the underlying population from which it was drawn. Describe also the setting, locations, and relevant dates, including periods of recruitment, exposure, follow-up, and data collection, if available.  b) Give the eligibility criteria, and the sources and methods of selection of participants.  c) Explain how the analyzed sample size was arrived at.  d) Describe measurement, quality and selection of genetic variants.  e) For each exposure, outcome and other relevant variables, describe methods of assessment and, in the case of diseases, the diagnostic criteria used.  f) Provide details of ethics committee approval and participant informed consent, if relevant. | Our MR analysis is based on a two-sample, two-step MR design and all analysis is based on existing GWAS resources. The GWAS data for the exposures are described in the “Immune cell traits GWAS data sources” section in the Methods, whereas the data for the Lung cancer-related phenotype outcomes are described in section “Lung cancer GWAS data sources”, whereas the data for the inflammatory cytokine mediators are described in section “Circulating inflammatory cytokines GWAS data source”. The eligibility criteria for each case are also described in those sections. For full details on the GWAS data used we refer and cite the original GWAS publications:  <https://gwas.mrcieu.ac.uk/>and <https://finngen.gitbook.io/documentation/v/r10/> and  (<https://data.bris.ac.uk/data/dataset/3g3i5smgghp0s2uvm1doflkx9x)>  Based on summary-level data, no ethical statement is required for this study. |
| 1. **Assumptions:** Explicitly state assumptions for the main analysis (e.g. relevance, exclusion, independence, homogeneity) as well assumptions for any additional or sensitivity analysis. | Described in the end of " IVs selection” section in the Methods. |
| 1. **Statistical methods main analysis**   Describe statistical methods and statistics used.  a) Describe how quantitative variables were handled in the analyses (i.e., scale, units, model).  b) Describe the process for identifying genetic variants and weights to be included in the  analyses (i.e, independence and model). Consider a flow diagram.  c) Describe the MR estimator, e.g. two-stage least squares, Wald ratio, and related statistics.  Detail the included covariates and, in case of two-sample MR, whether the same covariate set was used for adjustment in the two samples.  d) Explain how missing data were addressed.  e) If applicable, say how multiple testing was dealt with. | All main statistical methods are reported in the methods under the section ‘MR analyses’. These include the MR analysis methods and heterogeneity and pleiotropy exclusion methods. |
| 1. **Assessment of assumptions: Describe any methods used to assess the assumptions or justify their validity.** | We selected variants that were strongly associated with the exposures at GWAS significance to comply with assumption 1 and the threshold of significance were suggested by previous studies. We additionally used a large number of robust methods and sensitivity analyses, including F-statistic, using MR-presso/Cochran’s Q statistic, to probe into potential violations of assumptions 2 and 3. All methods are described under “MR analyses”. |
| 1. **Sensitivity analyses:** Describe any sensitivity analyses or additional analyses performed. | All sensitivity analyses are reported under the “MR analyses” section under the Methods. |
| 1. **Software and pre-registration**   a) Name statistical software and package(s), including version and settings used.  b) State whether the study protocol and details were pre-registered (as well as when and  where). | a) Reported in the 1st paragraph under ‘MR analyses’ section in the Methods  b) The study design is described in the end paragraph of "Introduction" section. |
| **Results** |  |
| 1. **Descriptive data**   a) Report the numbers of individuals at each stage of included studies and reasons for exclusion. Consider use of a flow-diagram.  b) Report summary statistics for phenotypic exposure(s), outcome(s) and other relevant variables (e.g. means, standard deviations, proportions).  c) If the data sources include meta-analyses of previous studies, provide the number of studies, their reported ancestry, if available, and assessments of heterogeneity across these studies. Consider using a supplementary table for each data source.  d) For two-sample Mendelian randomization:  i. Provide information on the similarity of the genetic variant-exposure associations between the exposure and outcome samples.  ii. Provide information on extent of sample overlap between the exposure and outcome data sources. | a) Information is given in the " Materials and methods" section.  b) We give the summary statistics for exposure in the Methods under “Immune cell traits GWAS data sources” section.  c) We give the summary statistics for each outcome in the Methods under “Lung cancer GWAS data sources section” in Table1.  d) We give the summary statistics for each mediators in the “Circulating inflammatory cytokines GWAS data source” section |
| 1. **Main results**   a) Report the associations between genetic variant and exposure, and between genetic variant and outcome, preferably on an interpretable scale (e.g. comparing 25th and 75th percentile of allele count or genetic risk score, if individual-level data available).  b) Report causal effect estimate between exposure and outcome, and the measures of uncertainty from the MR analysis. Use an intuitive scale, such as odds ratio, or relative  risk, per standard deviation difference.  c) If relevant, consider translating estimates of relative risk into absolute risk for a meaningful time-period.  d) Consider any plots to visualize results (e.g. forest plot, scatterplot of associations between genetic variants and outcome versus between genetic variants and exposure). | a) The associations between all IVs used in our analyses and our exposures and outcomes are reported in supplementary File 1-10.  b) Our results are reported in odds ratios per one standard unit increase of the exposure along with 95% confidence intervals throughout the results section.  c) We visualize results from the main MR result using a forest plot in Fig 4-7 |
| 1. **Assessment of assumptions**   a) Assess the validity of the assumptions.  b) Report any additional statistics (e.g., assessments of heterogeneity, such as I2, Q statistic). | a) To assess the validity of the assumptions we took the measures described in point 7; We selected variants that were strongly associated with the exposures at suggested GWAS significance i.e. P< 5×${10}^{-8}$ et al. to comply with assumption 1. B)We additionally used a large number of robust methods such as weighted median, MR-Egger, MR-Presso, contamination mixture and sensitivity analyses to probe into potential violations of assumption 3, mainly due to horizontal pleiotropy. All methods are described under “MR analyses” sections in the Methods. |
| 1. **Sensitivity and additional analyses**   a) Use sensitivity analyses to assess the robustness of the main results to violations of the assumptions.  b) Report results from other sensitivity analyses (e.g., replication study with different dataset, analyses of subgroups, validation of instrument(s), simulations, etc.).  c) Report any assessment of direction of causality (e.g., bidirectional MR).  d) When relevant, report and compare with estimates from non-MR analyses.  e) Consider any additional plots to visualize results (e.g., leave-one-out analyses). | a) and b) All sensitivity analyses performed are described under section “MR analyses” in the Methods. These include additional robust estimates for the univariable MR, alteration of the clumping threshold based on previous researches and variants included represented strong biological instruments. c) In the first step, we performed a two-sample bidirectional MR among exposure with outcome, exposure with mediator, and mediator with outcome. We excluded the MR results that exist a dual direction. d) We compared our results and experiment in vivo/vitro in the literatures in the discussion section. e) We used the forest plot and pie plot and so on to visualize our results. |
| **Discussion** |  |
| 1. **Key results** | We describe key results in the first paragraph of the discussion section. |
| 1. **Limitations**   Discuss limitations of the study, taking into account the validity of the MR assumptions, other sources of potential bias, and imprecision. Discuss both direction and magnitude of any potential bias, and any efforts to address them. | Limitations are reported in paragraph 4 of the Discussion. |
| 1. **Interpretations**   a) Give a cautious overall interpretation of results considering objectives and limitations.  Compare with results from other relevant studies.  b) Discuss underlying biological mechanisms that could be modelled by using the genetic  variants to assess the relationship between the exposure and the outcome.  c) Discuss whether the results have clinical or policy relevance, and whether interventions  could have the same size effect. | a) Reported in paragraphs 2&3 of the Discussion.  b) Discussion – paragraph 3 |
| 1. **Generalizability:** | We discuss potential caveats in terms of generalizability of discussion section in paragraph 4. |
| 1. **Funding:** | All sources of funding are reported. |
| 1. **Data and data sharing:** | All data used are publicly available which are available upon request from the websites. |
| 1. **Conflicts of Interest:** | All authors have declared conflicts of interest (none reported). |
